# Mapping biomass with remote sensing: a comparison of methods for the case study of Uganda

**DOI:** 10.1186/1750-0680-6-7

**Published:** 2011-10-07

**Authors:** Valerio Avitabile, Martin Herold, Matieu Henry, Christiane Schmullius

**Affiliations:** 1 Department of Environmental Science, Wageningen University, 6708 PB Wageningen, The Netherlands; 2 Institute of Geography, Friedrich-Schiller-University, Grietgasse 6, 07743 Jena, Germany; 3 IRD, UMR Eco&Sols, Montpellier SupAgro, Bat. 12, 2 place Viala, 34060 Montpellier Cedex 2, France; 4 Di.S.A.F.Ri, Università degli Studi della Tuscia, Via Camillo de Lellis, 01100, Viterbo, Italy; 5 AgroParisTech-ENGREF, GEEFT, 648 rue Jean-François Breton, BP 7355 - 34086 Montpellier Cedex 4, France

**Keywords:** forestry, global change, carbon, REDD+, sub-Saharan Africa, Land Cover, Landsat, MODIS, bio-energy

## Abstract

**Background:**

Assessing biomass is gaining increasing interest mainly for bioenergy, climate change research and mitigation activities, such as reducing emissions from deforestation and forest degradation and the role of conservation, sustainable management of forests and enhancement of forest carbon stocks in developing countries (REDD+). In response to these needs, a number of biomass/carbon maps have been recently produced using different approaches but the lack of comparable reference data limits their proper validation. The objectives of this study are to compare the available maps for Uganda and to understand the sources of variability in the estimation. Uganda was chosen as a case-study because it presents a reliable national biomass reference dataset.

**Results:**

The comparison of the biomass/carbon maps show strong disagreement between the products, with estimates of total aboveground biomass of Uganda ranging from 343 to 2201 Tg and different spatial distribution patterns. Compared to the reference map based on country-specific field data and a national Land Cover (LC) dataset (estimating 468 Tg), maps based on biome-average biomass values, such as the Intergovernmental Panel on Climate Change (IPCC) default values, and global LC datasets tend to strongly overestimate biomass availability of Uganda (ranging from 578 to 2201 Tg), while maps based on satellite data and regression models provide conservative estimates (ranging from 343 to 443 Tg). The comparison of the maps predictions with field data, upscaled to map resolution using LC data, is in accordance with the above findings. This study also demonstrates that the biomass estimates are primarily driven by the biomass reference data while the type of spatial maps used for their stratification has a smaller, but not negligible, impact. The differences in format, resolution and biomass definition used by the maps, as well as the fact that some datasets are not independent from the reference data to which they are compared, are considered in the interpretation of the results.

**Conclusions:**

The strong disagreement between existing products and the large impact of biomass reference data on the estimates indicate that the first, critical step to improve the accuracy of the biomass maps consists of the collection of accurate biomass field data for all relevant vegetation types. However, detailed and accurate spatial datasets are crucial to obtain accurate estimates at specific locations.

## Background

The accurate estimation of forest biomass is crucial for many applications, from monitoring fuelwood availability [1] to reducing uncertainties in global carbon (C) modeling [2-4]. Accurate biomass estimates are also required for the implementation of a reliable mechanism to reduce emissions from tropical deforestation and forest degradation (REDD+) under the United Nations Framework Convention on Climate Change (UNFCCC) [5]. While there is high interest in seeing such initiatives take form, monitoring forest biomass stocks and stock changes is identified as a key challenge for developing countries wishing to take part in the expected REDD+ mechanism [6,7].

Biomass stock over a certain area can be estimated based on area assessment of the different land uses and the associated biomass densities. In most developed countries land use area assessment is based on field measurement (e.g. using the cadastral system) and biomass stock is estimated from the national forest inventory. In tropical regions and particularly in sub-Saharan Africa, most of the countries do not have the technical and financial capacities to assess land use area through field measurement, and national forest inventories are often rare and outdated. As a consequence, the amount and distribution of tropical forest biomass is still highly uncertain [8,9].

In the last years new approaches using satellite observations and other spatial datasets were developed to optimally extrapolate field data over large areas [10,11] and a number of spatially explicit biomass and C datasets were produced for tropical regions [12-15,4] or with global coverage [16-18]. Given the relevance of this topic, space agencies are currently evaluating new satellite missions dedicated to biomass mapping, such as BIOMASS from ESA [19]. Nonetheless, a proper assessment of the accuracy of remotely sensed biomass maps is often limited by the lack of field validation datasets with comparable coverage and resolution, somehow hindering the operational use of the biomass products for national assessment of biomass and C stocks. Since maps based on global or regional datasets may not be tailored to country specific circumstances, their applicability at national scales needs to be better understood with appropriate case-studies.

In 1989, the government of Uganda established the National Biomass Study (NBS), a long term program aimed at the assessment of biomass resources and their dynamics at national level using country-specific data and methodology. The NBS is based on the combination of extensive biomass field measurements (over 5,000 plots), country-specific allometric equations and an ad-hoc land cover (LC) map [20]. Given its high quality, the NBS represents an optimal reference dataset to better understand the capabilities of existing biomass or C maps and related methodologies for national level applications. Specifically, the objectives of this study were to use the NBS dataset to: (1) compare existing biomass datasets; (2) quantify their accuracy; (3) assess the effect of different input data and methodologies on the biomass estimates.

## Methods

### Biomass maps

Six biomass or C stock maps, namely the Avitabile [21], Baccini [15], Drigo [12], Gibbs & Brown [14], Henry [4] and Reusch & Gibbs [17] maps, were compared to the NBS biomass map [20] for the area of Uganda (Table 1). While some maps were based on global and regional datasets, others were based on country-specific data. Datasets with resolution lower than 5 Km (e.g. [22,18]) were not included in the present analysis because they were considered too coarse in comparison with the limited extent of the country. While it is not possible to define with certainty the most accurate map because the true biomass values cannot be known, the NBS biomass map was considered as reference because based on the largest number of nation-specific field data and a widely accepted methodology.

**Table 1:** Main characteristics of the biomass and C maps used for the comparative study

Map	Coverage	Format	Spatial resolution/VMMU	Number of classes	Pool	Variable	Biomass data	Spatial data	Ancillary data	Period	Approach
NBS [20]	Uganda	Vector	4 - 50 ha	48	Aboveground	Biomass	NBS Field data	NBS LC map	NBS Ecozone map	circa-2000	CA

Avitabile [21]	Uganda	Raster	30 × 30 m	376	Aboveground	Biomass	subset of NBS Field data	Landsat	None	1999-2003	DR+

Baccini [15]	Tropical Africa	Raster	1 × 1 Km	343	Aboveground	Biomass	Field data	MODIS	None	2000-2003	DR

Drigo [12]	Eastern Africa	Vector	40 - 120 ha	143	Aboveground	Biomass	Biome average	Africover	FAO GEZ	circa-2000	CA

Gibbs & Brown [14]	Tropical Africa	Raster	5 × 5 Km	176	Above and belowground	Biomass	Biome average	GLC2000	CIESIN Population	2000	CA

Henry [4]	sub-Saharan Africa	Raster	300 × 300 m	346	Aboveground	Carbon	Field data	Globcover	FAO GEZ, MODIS VCF	2005	CA+

Reusch & Gibbs [17]	Global	Raster	1 × 1 Km	14	Above and belowground	Carbon	IPCC Biome average	GLC2000	FAO GEZ, Frontier forest map	2000	CA

The spatial resolution of vector-based maps is given by a Variable Minimum Mapping Unit (VMMU), which is class-specific. The NBS map used in this study was derived from the NBS LC map by applying to each stratum its respective average biomass density reported in Drichi [21]. The spatial datasets used by the biomass/C maps included the Africover LC map [36], the Global Land Cover map for the year 2000 (GLC2000) [37], the Globcover 2005 map [38], the Food and Agriculture Organization (FAO) Global Ecological Zone (GEZ) map [30], the MODIS Vegetation Continuous Field (VCF) tree cover products [39], the CIESIN's Gridded Population of the World dataset [40] and the Frontier Forest map [41]. The number of biomass classes of the maps with continuous values was calculated after their conversion to integer values (i.e. classes of 1 Mg ha^-1 ^interval).

The approaches used by the maps to relate spatial data to ground observations were classified using the scheme presented by Goetz et al. [10]: (1) Stratify & Multiply (SM), where satellite data are used to derive a thematic map and the field biomass data within each class are averaged; (2) Combine & Assign (CA), where satellite and other spatial datasets are integrated to derive a thematic map with finer-grained strata and the field data within each stratum are averaged; (3) Direct Remote Sensing (DR), where satellite data are directly converted to biomass density using classification techniques (e.g. neural network, regression trees). Linear regression or model inversion techniques are also employed, especially in combination with active remote sensing sensors [13,23].

The approach used by the Avitabile map is an extension of the DR approach (DR+) because it integrates satellite with LC data using a statistical model. Similarly, the Henry map is based on an extended CA approach (CA+) using a Monte Carlo procedure and satellite inputs in combination with categorical data (i.e. LC map) to obtain almost continuous biomass estimates.

### Maps pre-processing

The datasets were standardized with regard to measurement variable, reference system, spatial coverage and spatial resolution. Aboveground biomass (AGB) was used as a common measurement unit and the carbon maps were converted into AGB using a carbon ratio of 0.47 for the Reusch & Gibbs map and 0.5 for the Gibbs & Brown and Henry maps. When the maps reported total (above and belowground) biomass or carbon stock, the aboveground component was calculated using root-to-shoot ratios, namely the IPCC [24] ratios for the Reusch & Gibbs map and the Gaston et al. [25] ratios for the Gibbs & Brown map. Before the comparison of each map to the NBS, the two datasets were projected to a common reference system and, in order to minimize geolocation errors, higher resolution datasets were re-projected to the reference system of the dataset at lower resolution. The maps were co-registered if necessary and then aggregated to a common resolution on the basis of the following procedure, depending on the nature (vector, raster) and native resolution of the datasets. When the maps to be compared were both raster-based, the dataset at higher resolution was aggregated and resampled to the grid and pixel size of the dataset at lower resolution. When both maps were vector-based, the map with smaller Minimum Mapping Unit (MMU) (i.e. higher spatial detail) was first converted to high-resolution (30 m) raster and then aggregated within the polygons of the map with larger MMU. When the comparison was done between a vector-based and a raster-based map with lower resolution, the vector-based map was converted to high-resolution raster and then aggregated to the cell size and grid of the low-resolution raster. Instead, when the raster-based map had a higher resolution than the vector-based map, the raster was aggregated within the vector polygons and the comparison was performed at polygon level. The reference NBS map, which was vector-based with a variable MMU ranging from 0.04 Km^2 ^for forests to 0.5 Km^2 ^for grasslands, was considered as having higher resolution than the raster maps with cell size ≥ 1 Km but lower resolution than the raster maps with cell size ≤ 300 m.

### Comparison of maps

The level of agreement of the biomass maps with the NBS map was assessed by comparing total and spatial distribution of the biomass estimates. Spatial similarity was assessed on the basis of difference maps, the Fuzzy Numerical (FN) index, FN maps and variograms.

The difference maps, obtained by subtracting corresponding map cells, represent the difference of the map estimates at pixel level while the FN index [26,27] measures the similarity of spatial patterns between two numerical raster maps. The FN index, ranging between 0 (fully distinct maps) and 1 (fully identical maps), is computed as the average of the numerical similarity *s *between each pair of corresponding cells (*a *and *b*) in the two maps, which in turn is computed cell-by-cell as follows:

(1) $$\text{s}\left(\text{a},\text{b}\right)=1-\frac{|a-b|}{\max\left(|a|,|b|\right)}$$

where the cell values (*a *and *b*) are re-computed considering the neighboring cells within a specified window. The FN index provides a single measure of the maps' overall agreement while the FN map represents the numerical similarity at pixel level.

The variograms of the biomass maps were computed to represent and compare the spatial variation of areas with homogeneous biomass density in the datasets [28,29]. The map variograms were also compared with the variogram derived from the field plots in order to identify the map most able to maintain the spatial variation represented by the field data. Since the variograms are sensitive to the spatial resolution of the dataset [28], the maps were converted to raster format at 1 Km resolution before the analysis and the Gibbs & Brown map was excluded because of its lower native resolution. Water bodies were masked before the FN and variogram analysis.

In order to investigate the effect of spatial resolution on the maps similarity, the spatial statistics were computed at the map native resolution and, after aggregation through averaging, at other resolutions ranging from 1 to 50 Km.

### Comparison of maps with field data

The accuracy of the biomass maps was assessed using available NBS field data. This validation dataset consisted of 3510 field plots with a spatial extent of 50 × 50 m systematically located throughout the country at 5 × 10 Km distance and measured between 1995 and 2005. Clusters of 1 to 5 plots were located at each grid intersection separated by a distance of 300 m. The field plots were up-scaled to the resolution of the biomass maps using the following procedure. For vector-based maps, the biomass values of the plots located within each polygon were averaged. For raster-based maps, the plots located within each pixel were area-weighted averaged on the basis of the NBS LC map (i.e. weighting the plots according to the fraction of the corresponding LC polygon area within each pixel) using only the pixels where the field plots represented, through LC polygons, at least 90% of the pixel area. Since the comparison is affected by the map resolution, the Avitabile and Henry maps were aggregated to 1 Km resolution before the analysis.

The upscaled plot values were then compared with the estimates of the corresponding unit (pixel or polygon) of the biomass maps. Since categorical maps (i.e. maps based on the SM or CA approach) attribute an average value to the map units belonging to the same class, the average of all upscaled field plots located within the same biomass class was also compared with the class value itself. Lastly, in order to test the assumption that 1 plot (area of 0.0025 Km^2^) may not be comparable with the larger map units (area of 1 Km^2 ^for most datasets), the comparison statistics were re-computed selecting only the map units with at least 2 field plots.

### Comparison of biomass reference values and spatial data

The biomass maps considered in this study were obtained by combining biomass reference values with spatial datasets. In order to separate and quantify the impact of these two components on the map estimates, the same set of biomass reference values was applied to different spatial maps, and viceversa. Specifically, the most detailed biomass reference values (the NBS plots) and then the most general values (the IPCC Tier 1 values) were applied to the NBS LC map, to the GLC2000 map stratified by the Food and Agriculture Organization (FAO) Global Ecological Zone (GEZ) map [30] (as in the Reusch & Gibbs map) and to the Globcover map stratified by the FAO GEZ map (as in the Henry map). The NBS plots were also applied to Landsat data in the Avitabile map.

## Results

The comparison results are to be interpreted keeping in consideration that the Avitabile and Baccini maps were not independent from the reference data. Specifically, the Avitabile map used the NBS field plots and the NBS LC map to estimate biomass density while the Baccini map used the NBS biomass map to derive training data. On the other hand, the Drigo, Gibbs & Brown, Henry and Reusch & Gibbs maps were independent from the reference data and the comparison results represent an independent validation of their performance for the area of Uganda. However, the results were partially affected by differences in map formats and resolutions. For example, coarser maps (e.g. Gibbs & Brown, Drigo) were favored in the comparison because the similarity between the datasets tended to increase at lower resolution (see below).

### Comparison of maps

#### Total aboveground biomass of Uganda

**Figure 1:**
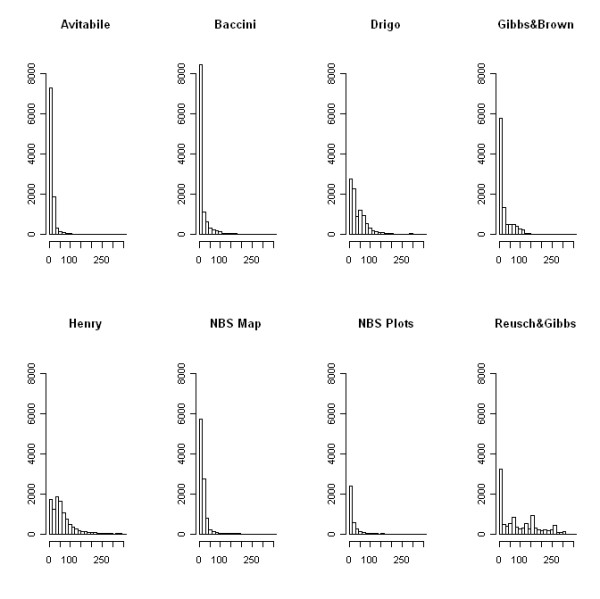
**Histograms of the biomass maps and NBS field plots**. The histograms represent the frequency (i.e. number of occurrence) (y axis) for each AGB class (x axis). AGB is reported in Mg ha^-1^. The histograms are derived from maps aggregated at the resolution of the coarser map (5 × 5 Km) for consistent representation of frequency values.

The comparison of biomass stock of Uganda revealed strong disagreement between the remote sensing products, with estimates of total AGB ranging from 343 to 2201 Tg (Table 2). The Baccini map provided the closest estimate (443 Tg) to the NBS reference value (468 Tg) while the Reusch & Gibbs map provided the most different value (2201 Tg). Estimates from maps based on the DR approach were conservative (i.e. negative bias) while maps based on the CA approach provided higher values than the NBS (i.e. positive bias). The map histograms (Figure 1) showed large differences among the distribution of the biomass estimates, with the Avitabile, Baccini and NBS maps as well as the NBS field plots being concentrated at low values (< 100 Mg ha^-1^) and the Drigo, Gibbs & Brown, Henry and Reusch & Gibbs maps presenting higher frequencies at higher values.

**Table 2:** Total and mean AGB of Uganda for different biomass maps

Map	Total AGB (Tg)	Mean AGB (Mg/ha)	Bias (Mg/ha)
Avitabile	343	14.2	-5.2
Baccini	443	18.4	-1.0
NBS	468	19.4	-
Gibbs & Brown	579	24.0	4.6
Drigo	1191	49.3	29.9
Henry	1550	64.2	44.8
Reusch & Gibbs	2201	91.1	71.7

Houghton [42]/DeFries [43]^a^	674	27.9	8.5
Brown [44]/Achard [45,46]^a^	744	30.8	11.4
Olson [47]/Gibbs [16]^a^	832	34.5	15.1
IPCC [24]^a^	1921	79.5	60.2

The map bias is relative to the NBS value. The values for the maps indicated with the symbol (^a^) are derived from [11]

#### Other estimates of Uganda's biomass

Similar level of disagreement among AGB estimates of Uganda, as the tendency of maps based on biome average values and coarse spatial datasets to overestimate this parameter, was observed in Gibbs et al. [11]. This study estimated total above and belowground C stock for several tropical countries combining four sets of biomass reference values with the GLC2000 map stratified by the FAO GEZ map. After converting the Gibbs et al. [11] values for Uganda to AGB by applying a biomass conversion factor of 0.47 and the root-to-shoot ratio for rainforest (the most common ecozone of Uganda) of 0.37 [24], the estimates were between 674 and 1921 Tg, within the range found in the present comparison but consistently above the NBS reference value (Table 2). Higher AGB values were obtained using root-to-shoot ratios for the other ecozones present in Uganda.

High variability of estimates of forest biomass was also noticed comparing the FAO Forest Resource Assessment (FRA) statistics for Uganda for the year 2000, which indicate 681 Tg in the FRA 2000 Report [31] and only 244 Tg in the FRA 2005 Report [32]. The difference, due to the adoption of the NBS data in the FRA 2005 Report, indicates the sensitivity of this parameter to the input data and methodology used to estimate it. The value reported in the FRA 2005 refers to forest AGB and is about half of the NBS value due to the fact that in Uganda large amount of biomass are stored in areas outside forest land.

#### Spatial distribution of biomass

**Figure 2:**
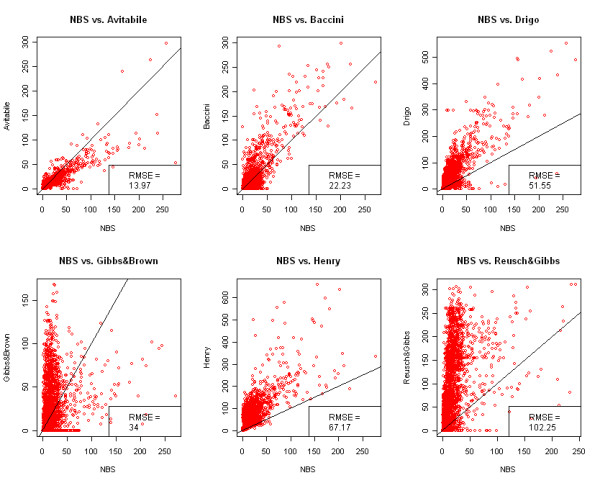
**Comparison of AGB values between the NBS and the other biomass maps**. The comparison is performed at pixel level and the results are reported for the maps aggregated at 10 Km resolution for graphical reasons. AGB is reported in Mg ha^-1^. RMSE: root mean square error.

**Figure 3:**
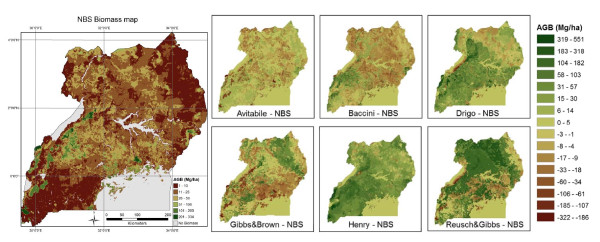
**NBS reference biomass map (left) and difference of AGB values between the biomass maps and the NBS map (right)**. The difference maps, obtained by subtracting the corresponding map pixels, indicate overestimation with positive values (in green) and underestimation with negative values (in brown) in comparison to the NBS map. The NBS map is modified from [20]. AGB is reported in Mg ha^-1^.

**Figure 4:**
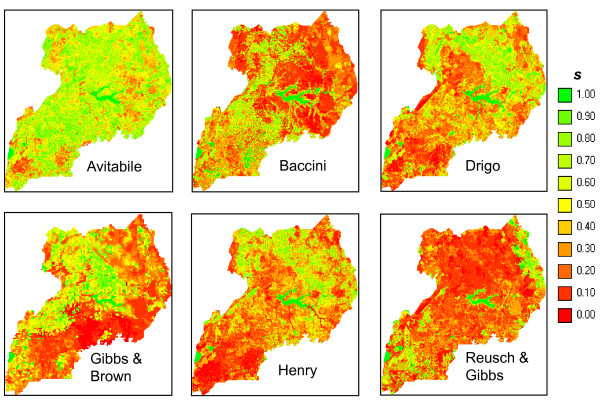
**Fuzzy Numerical maps**. The Fuzzy Numerical maps represent the spatial distribution of the numerical similarity (*s*) between the biomass maps and the NBS map, ranging from 0 (fully distinct) to 1 (fully identical).

**Figure 5:**
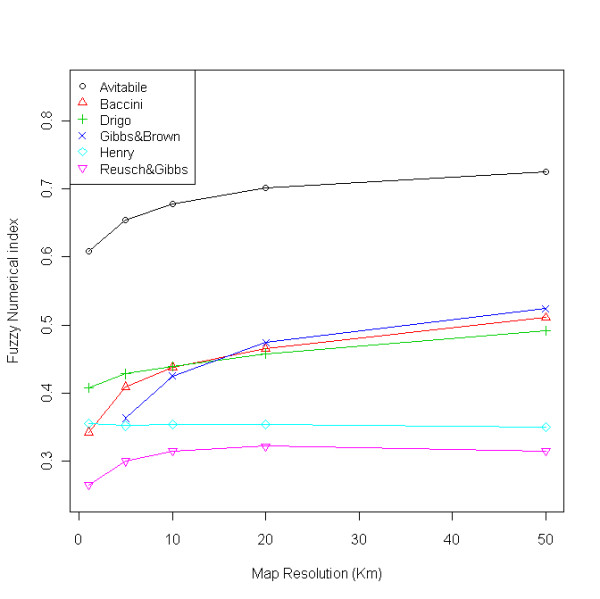
**Fuzzy Numerical index**. The Fuzzy Numerical index represents the mean similarity between the biomass maps and the NBS map computed at different spatial resolutions (1 to 50 Km).

Differently from summary statistics (e.g. total biomass) where positive and negative differences may compensate each other, spatial comparison of the maps reveals their similarity at local level. The scatterplots (Figure 2), difference maps (Figure 3) and FN maps (Figure 4) showed that the Reusch & Gibbs map strongly overestimated AGB distribution over most of the country and especially at low biomass values. The Gibbs & Brown map overestimated AGB in the northern part of Uganda and underestimated it in the south-eastern region. Conversely, both the Drigo and Henry maps overestimated AGB in the southern region and at high biomass values, and presented estimates similar to the NBS in the northern region. The spatial analysis of the Baccini map, which total AGB was very similar to the NBS value, revealed the presence of local differences, with overestimation in the western region counterbalanced by underestimation in the center-eastern region. The Avitabile map presented high spatial agreement with the NBS map and provided lower biomass densities over most of the country, with higher differences in the forest areas. The FN index (Figure 5) confirmed these findings and showed that the spatial agreement between the maps usually increased at lower spatial resolution.

**Figure 6:**
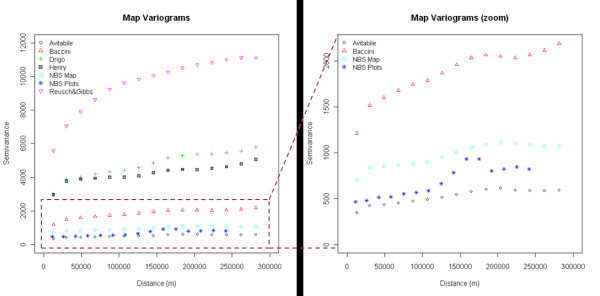
**Variograms of the biomass maps and NBS field plots**. The right figure shows a zoom of the variograms for semivariance values between 0 and 2500.

The comparison of variograms (Figure 6) indicated a large variability in the variance of the datasets. The field plot variogram was best approximated by the Avitabile and NBS variograms, suggesting that these two maps best maintained the biomass spatial variation represented by the field data. Instead, the other maps presented higher semivariance, indicating larger variations in biomass predictions compared to those observed from the field data at corresponding distances. Variogram behavior was related to the stratification approach employed by the maps and showed that maps based on coarse stratification layers and biome average values (i.e. Reusch & Gibbs, Drigo) could not capture the high biomass spatial variability represented by the field plots but mapped large homogeneous areas or few, distinct biomass classes. On the contrary, maps based on a regression approach or several detailed strata (i.e. Avitabile, NBS) identified small biomass areas with a continuum of values, representing more closely the spatial characteristics of the field data. The variogram of the Baccini map, based on a regression approach but with coarse resolution, showed an intermediate behavior while the Henry map, despite its higher resolution and large number of biomass classes, represented a biomass spatial variation not matching with that of the field plots.

#### Comparison of maps with field data

The comparison of the biomass maps with the field plots (Table 3) confirmed the large differences among the datasets.

**Table 3:** Comparison of the biomass maps with the NBS field data

	All plots			Plots ≥ 2		
	
Map	N	Bias (Mg/ha)	RMSE (Mg/ha)	N	Bias (Mg/ha)	RMSE (Mg/ha)
Avitabile	850	-2.9	17.7	173	-1.7	13.6
Baccini	888	-4.8	24.7	184	-7.5	23.5
Drigo	985	39.3	57.2	664	38.8	54.0
Gibbs&Brown	92	9.2	30.4	31	6.1	28.0
Henry	878	51.1	62.2	183	57.1	69.9
NBS	1129	1.1	24.1	693	0.9	19.5
Reusch&Gibbs	778	35.5	66.7	160	31.5	65.1

The comparison is performed using all map units with at least 1 field plot ("All plots", left column) and selecting only the map units with 2 or more field plots ("Plots ≥ 2", right column). N is the number of map units used for the comparison

The error estimate was higher for the Reusch & Gibbs, Henry, Drigo and Gibbs & Brown maps, which presented a Root Mean Square Error (RMSE) equal to 66.7, 62.2, 57.2 and 30.4 Mg ha^-1 ^respectively. Since the reference field data were independent from these maps, the error estimates quantify the map accuracies for the area of Uganda. However, the RMSEs were reduced by the skewed distribution of the field data (see Figure 1), which focused the comparison on low biomass areas where prediction errors tended to be smaller than those in high biomass areas.

The other three maps, directly or indirectly related to the field data, presented lower error estimates.

The RMSE of the Avitabile map aggregated at 1 Km resolution (17.7 Mg ha^-1^) was comparable with the value computed at the map's native resolution using independent NBS field plots (15 Mg ha^-1^) [21]. Similarly, the RMSE of the NBS map (24.1 Mg ha^-1^) was comparable with the mean standard deviation of the biomass strata (16.9 Mg ha^-1^) reported by [20]. Instead, the RMSE of the Baccini map for Uganda (24.7 Mg ha^-1^) was lower than the value reported by [15] for tropical Africa computed using independent reference data (50.5 Mg ha^-1^), possibly because in the present analysis the map was indirectly related to the reference data.

When the comparison was performed selecting only the map units (pixels or polygons) with 2 or more field plots, there was only a small increase in the map accuracies, while the number of units available for the comparison decreased considerably (Table 3). When the comparison was performed for biomass classes (as defined above) instead of map units, the RMSE values were higher (Table 4), possibly because the aggregation in classes reduced the effect of the skewed distribution of the field data.

**Table 4:** Comparison of the biomass maps with the NBS field data by biomass classes

	All plots			Plots ≥ 2		
	
Map	N	Bias (Mg/ha)	RMSE (Mg/ha)	N	Bias (Mg/ha)	RMSE (Mg/ha)
Avitabile	52	-0.9	24.7	45	-0.9	20.0
Baccini	75	26.7	45.0	56	14.5	31.5
Drigo	94	47.7	64.5	90	45.5	61.5
Gibbs&Brown	36	36.2	47.9	14	9.3	34.0
Henry	163	75.4	88.5	130	67.4	79.6
NBS	34	8.6	28.7	33	7.8	28.5
Reusch&Gibbs	11	70.7	104.3	11	70.7	104.3

The comparison is performed using all map units with at least 1 field plot ("All plots", left column) and selecting only the map units with 2 or more field plots ("Plots ≥ 2", right column). N is the number of biomass classes with field plots identified in each map

#### Comparison of biomass reference values and spatial data

The comparison of AGB estimates obtained using different combinations of the input data showed that applying different biomass reference values to the same spatial map caused very large variations in the biomass estimates (219 - 504%) (Table 5). On the contrary, using different spatial maps to stratify a set of biomass reference value caused much smaller variations (20 - 33%) (Table 6). However, the spatial datasets influenced the distribution of the estimates and, according to the FN index, the overall spatial agreement of the biomass maps with the NBS reference map decreased using global LC datasets instead of Landsat data or the NBS LC map (Table 6).

**Table 5:** Total AGB using different combinations of spatial and biomass data

Spatial data	Biomass data	Total AGB (Tg)	Difference (%)
NBS LC	NBS plots	468	-
	IPCC Tier1	1,492	219%

GLC2000 + FAO GEZ	NBS plots	377	-
	IPCC Tier1	2,234	493%

GlobCover + FAO GEZ	NBS plots	363	-
	IPCC Tier1	2,191	504%

The right column reports the percentage difference of total AGB using different biomass reference data for each spatial dataset

**Table 6:** Total AGB using different combinations of biomass and spatial data

Biomass data	Spatial data	Total AGB (Tg)	Difference (%)	Fuzzy Numerical
NBS plots	NBS LC	468^a^	-	-
	GLC2000+FAO GEZ	377	-19.5%	0.568
	GlobCover+FAO GEZ	363	-22.5%	0.565
	Landsat	343^b^	-26.7%	0.608

IPCC Tier1	GLC2000+FAO GEZ	2,234	-	0.265
	GlobCover+FAO GEZ	2,191	-1.9%	0.292
	NBS LC	1,492	-33.2%	0.410

The values indicated with the symbol (^a^) and (^b^) are derived from [20] and [21], respectively. The table also reports the percentage difference of total AGB using different spatial dataset for each biomass reference data and the Fuzzy Numerical index. The Fuzzy Numerical index is computed by comparing each map with the NBS reference map (NBS plots + NBS LC) at 1 Km resolution

The results also indicated that the use of the IPCC Tier 1 values in combination with different spatial maps always over-estimated AGB of Uganda (Table 5). The comparison of the biomass estimates separately per vegetation type revealed that most of the overestimation was due to the IPCC reference values for forest (ranging from 115 to 310 Mg ha^-1^), which were significantly higher than the corresponding NBS values (ranging from 35 to 223 Mg ha^-1^) and, when applied to the NBS LC map, estimated forest biomass to 1279 Tg while this was only 294 Tg according to the NBS (Table 7).

**Table 7:** Comparison of biomass and area statistics for the main LC classes of Uganda

	AGB Ref. values (Mg/ha)		Total AGB (Tg)		Area (Km^2 ^× 1000)	
**LC class**	**NBS Plots**	**IPCC Tier1**	**NBS Plots + NBS LC**	**IPCC Tier1 + NBS LC**	**NBS LC**	**GLC2000**

Forest	35 - 223	115 - 310	294	1279	49.3	67.3
Shrubland	14	70	14	98	14.2	49.2
Grassland	8	2 - 6	47	31	51.2	15.8
Agricolture	11	10	112	84	84.7	70.7

For each LC class, the comparison is performed between the NBS and the IPCC Tier 1 biomass reference values (left columns), between the total AGB estimates of Uganda obtained by applying the NBS and IPCC reference values to the NBS LC map (central columns), and between the area mapped by the NBS LC and the GLC2000 maps (right columns)

In addition, the comparison of the LC maps revealed large differences among the datasets. Specifically, the GLC2000 mapped larger areas of forest and shrubland and smaller extents of grassland and agriculture in comparison to the NBS (Table 7). Therefore, by using the IPCC Tier 1 values in combination with the GLC2000 map (as in the Reusch & Gibbs map), the higher biomass reference values for forest and shrubland were applied to larger area coverage of these two classes, causing the strong overestimation of AGB stock found in the Reusch & Gibbs map.

## Discussions

The comparison of six biomass and C stock maps with the NBS reference dataset revealed large differences regarding estimates of total AGB of Uganda and its spatial distribution. These differences were related to the biomass reference data (biome average values versus field data), the spatial datasets (global versus national maps or satellite data) and the statistical approach (averaging versus regression models) employed by the maps.

The biomass reference data were responsible for most of the variability in the AGB estimates (Table 5). Maps based on biome average biomass values (i.e. Reusch & Gibbs) and averages from forest inventories acquired in several countries (i.e. Drigo, Gibbs & Brown) agreed less with the reference dataset than maps based on national forest inventory data, strongly overestimating the country's AGB stock. This was due to the fact that biome average values, representative of large areas and broad vegetation types, were applied to areas of Uganda that diverge from the average biome conditions. For example, the IPCC Tier 1 value of 260 Mg ha^-1 ^for AGB in forests in the tropical moist deciduous ecozone was not appropriate to the woodlands in northern Uganda, located in an area much drier and with lower tree density and dimension than in the average ecozone condition, where the NBS reference value was 35 Mg ha^-1^. In addition, while biomass density in mature forest and at specific locations can reach higher values than the average NBS values [23], applying default AGB values to 1 Km resolution maps is particularly not appropriate in a country like Uganda, which is characterized by a highly fragmented landscape. However, while the use of biome average values overestimated the total AGB of Uganda, in some cases their application to local level underestimated AGB density (see Figure 3), which in terms of C assessment may cause underestimation of emissions from deforestation occurring in forests with more than average biomass density [2,8].

Regarding the spatial datasets, maps based on global LC datasets agreed less with the reference data than maps based on national LC or satellite data. Global datasets represent the distribution of the main LC types at regional level using a limited number of classes and, as a consequence of the small thematic detail, the variability of a biophysical parameter as biomass within the LC classes is usually large. For example, in the GLC2000 dataset all areas dominated by trees (> 40% cover) are classified as forest, but it can be expected that even after their stratification by ecological regions, the biomass density within a forest type varies considerably according to tree size, density and specific conditions related to local climate and land use history. Instead, national LC maps identified different forest types that allowed reducing the variability in the AGB strata. Satellite data were also able to identify strata with low AGB variability and, in comparison with LC maps, presented the advantage of higher spatial resolution, higher thematic content (i.e. continuous values) and were not affected by ambiguities in class definition or propagation of LC errors in the biomass estimates.

Regarding the statistical approach, biomass estimates derived from averaging methods (CA approach) agreed less with the reference data than predictions based on regression models (DR approach). While this result was clearly affected by the fact that the Avitabile and the Baccini maps (DR approach) were not independent from the reference datasets, similar conclusions were reached by Goetz et al. [10] on the basis of independent comparison data. Using lidar metrics closely related to AGB density, Goetz et al. showed that in central Africa the Baccini map (DR approach) had a narrower range of variability of lidar values within each AGB class and hence a smaller uncertainty for any AGB estimate than the Gibbs & Brown map (CA approach). It is important to note that these results are due to the input data employed by the CA maps (i.e. biome average values and coarse spatial maps) more than to the approach itself. Ultimately, the quality of a biomass map depends on the input data used to generate it. However, the factors reducing the map accuracy were strictly associated with the approach: for instance, the DR approach could hardly be developed on the basis of few average biomass values, and it employs satellite data that are certain to have higher spatial and thematic resolutions than any derived map. In addition, since all the map units belonging to the same class receive the same biomass value, the averaging approach cannot explain the intra-class variability, which can be large when the strata do not accurately reflect the biomass distribution. Instead, maps based on regression models provide continuous estimates that can describe the full range of biomass variability. Moreover, while the categorical data (such as LC) that are used to identify the strata can only represent the dominant class within a certain unit (unless there is no dominant class and the unit is defined as a "mosaic"), the continuous nature of remotely sensed surface reflectance accounts also for the minor components, but this capability is limited by the complexity of retrieving biomass from a mixed signal.

Maps based on the DR approach showed the tendency to underestimate biomass density, which is mainly a consequence of three factors related to the use of optical data and decision tree models (see also [21]). First, the optical signal tends to saturate in closed canopy forests resulting in the tendency to underestimate areas with high biomass densities. Second, in order to minimize atmospheric effects (i.e. cloud coverage, haze) satellite images are often acquired during the dry season when deciduous vegetation is usually without leaves and may be confounded with low-vegetation areas. Third, by averaging the data within each terminal node, decision tree models intrinsically underestimate at high values (and overestimate at low values).

We note that the differences between the approaches tend to reduce when the maps based on the CA approach identify several strata with high spatial and thematic resolution, and when the continuous AGB estimates provided by the DR approach are aggregated in classes to achieve satisfactorily accuracies.

The differences among the maps were also affected by the lack of a common definition of AGB. Some maps (NBS and Avitabile) refer to air-dry biomass while others refer to oven-dry biomass (Henry, Reusch & Gibbs) or do not provide this information (Baccini, Drigo, Gibbs & Brown) Similarly, there are differences regarding the minimum diameter and the inclusion or exclusion of dead trees, non-woody plants (herbaceous plants, lianas) or non-woody components (foliage, seeds). However, considering that oven-dry biomass is equivalent to about 80% of air-dry biomass [33] and that in some ecosystems tree biomass can represent more than 95% of the total AGB [4], the contribution of different AGB definition can only account for a limited portion of the large differences present among the maps.

The comparison of the biomass maps with ground reference data confirmed the above findings but also demonstrated the difficulty to perform a consistent validation of remote sensing products. Compatible maps as well as an accurate, representative and comparable field dataset are required to obtain comparable and reliable estimates of map accuracy, but such datasets are usually not available. In the present analysis the validation results were affected by differences in the format (vector, raster), spatial resolution and biomass definition used by the maps, by the skewed distribution of the field data and, most importantly, by the fact that some datasets were not independent from the testing data.

Lastly, this study demonstrates that the AGB estimates were primarily driven by the biomass reference values while the type of spatial datasets used for their stratification had a smaller, but not negligible, impact. The results highlight the importance of the applicability of biomass reference values to the study area but also indicate that the resolution and accuracy of the spatial data are still critical to obtain reliable AGB estimates at local level, which are necessary for land management or estimating emissions from deforestation at specific locations.

## Conclusions

In order to better understand the reliability of existing biomass and C stock products, we compared six maps with a reference dataset for the case study of Uganda. The comparison revealed very large differences among the datasets and indicated that maps employing the CA approach in combination with biome average values (as the IPCC Tier 1 values) and global LC datasets strongly overestimated the biomass stock. Instead, more reliable estimates were obtained using country-specific field data (i.e. forest inventories) in combination with satellite data and/or national LC and ancillary information. Maps based on satellite data were able to provide continuous and spatially detailed biomass estimates. These maps tended towards conservative estimates mainly as a consequence of the processing techniques and the saturation of the satellite signal at high biomass values. The comparison with ground reference data confirmed these findings but the validation results were not entirely comparable as they were affected by several factors. The larger impact of biomass reference data than spatial maps on AGB estimates indicates that the first critical step to improve the accuracy of the biomass maps consists of the collection of accurate biomass field data for all relevant vegetation types. However, detailed and accurate spatial datasets are crucial to obtain accurate estimates at specific locations and to correctly quantify emissions from deforestation as required for REDD+ actions. The acquisition of field datasets comparable with the remote sensing products is also necessary for their proper validation. With new remote sensing based maps of forest parameters recently produced over large areas, such as the global forest canopy height map [34] or the pan-tropical forest carbon stock map [35], the development of reliable reference datasets and appropriate comparison techniques is crucial to better understand the capabilities and limitations of such datasets.

## Competing interests

The authors declare that they have no competing interests.

## Authors' contributions

VA drafted the manuscript, developed the methodological approach and carried out the data analysis; VA, MHer, MHen and CS conceived the study, MHer, MHen and CS contributed to develop the methodological approach, MHer and MHen contributed to the manuscript. All authors read and approved the manuscript.

## References

[B1] Masera O Ghilardi A Drigo R Trossero MA WISDOM: A GIS-based supply demand mapping tool for woodfuel management Biomass & Bioenergy 2006 30 618 637 10.1016/j.biombioe.2006.01.00621987797

[B2] Houghton RA Aboveground forest biomass and the global carbon balance Global Change Biology 2005 11 945 958 10.1111/j.1365-2486.2005.00955.x

[B3] Le Quere C Raupach MR Canadell JG Marland G Bopp L Ciais P Conway TJ Doney SC Feely RA Foster P Friedlingstein P Gurney K Houghton RA House JI Huntingford C Levy PE Lomas MR Majkut J Metzl N Ometto JP Peters GP Prentice IC Randerson JT Running SW Sarmiento JL Schuster U Sitch S Takahashi T Viovy N van der Werf GR Woodward FI Trends in the sources and sinks of carbon dioxide Nature Geoscience 2009 2 831 836 10.1038/ngeo689

[B4] Henry M Carbon stocks and dynamics in Sub Saharan Africa PhD Thesis 2010 University of Tuscia, AgroParisTech/ENGREF http://www5.montpellier.inra.fr/ecosols/Recherche/these_et_hdr/these_de_matieu_henry

[B5] UNFCCC Draft decision -/CP.16 Outcome of the work of the Ad Hoc Working Group on long-term Cooperative Action under the Convention--policy approaches and positive incentives on issues relating to reducing emissions from deforestation and forest degradation in developing countries; and the role of conservation, sustainable management of forests and enhancement of forest carbon stocks in developing countries 2010

[B6] Herold M Skutsch M Monitoring, reporting and verification for national REDD+ programmes: two proposals Environmental Research Letters 2011 6

[B7] Henry M Maniatis D Gitz V Huberman D Valentini R Implementation of REDD plus in sub-Saharan Africa: state of knowledge, challenges and opportunities Environment and Development Economics 2011 16 381 404 10.1017/S1355770X11000155

[B8] Houghton RA Hackler JL Emissions of carbon from land use change in sub-Saharan Africa Journal of Geophysical Research-Biogeosciences 2006 111

[B9] Waggoner PE Forest Inventories: Discrepancies and Uncertainties "The World's Forests: Design and Implementation of Effective Measurement and Monitoring" 2009 Washington, DC: Resources for the Future

[B10] Goetz SJ Baccini A Laporte NT Johns T Walker W Kellndorfer J Houghton RA Sun M Mapping and monitoring carbon stocks with satellite observations: a comparison of methods Carbon Balance Management 2009 4 2 10.1186/1750-0680-4-2 PMC2667409 19320965

[B11] Gibbs HK Brown S Niles JO Foley JA Monitoring and estimating tropical forest carbon stocks: making REDD a reality Environmental Research Letters 2007 2

[B12] Drigo R WISDOM - East Africa. Woodfuel Integrated Supply/Demand Overview Mapping (WISDOM) Methodology. Spatial woodfuel production and consumption analysis of selected African countries Wood Energy Working Paper: FAO Forestry Department 2006 http://www.fao.org/docrep/009/j8227e/j8227e00.HTM

[B13] Saatchi SS Houghton RA Alvala R Soares JV Yu Y Distribution of aboveground live biomass in the Amazon basin Global Change Biology 2007 13 816 837 10.1111/j.1365-2486.2007.01323.x

[B14] Gibbs HK Brown S Geographical Distribution of Woody Biomass Carbon in Tropical Africa: An Updated Database for 2000 2007 Oak Ridge, Tennessee: Carbon Dioxide Information Center, Oak Ridge National Laboratory

[B15] Baccini A Laporte N Goetz SJ Sun M Dong H A first map of tropical Africa's above-ground biomass derived from satellite imagery Environmental Research Letters 2008 3 045011 10.1088/1748-9326/3/4/045011

[B16] Gibbs HK Olson's Major World Ecosytem Complexes Ranked by Carbon in Live Vegetation: An Updated Database Using the GLC2000 Land Cover Product 2006 Oak Ridge, Tennessee: Carbon Dioxide Information Center, Oak Ridge National Laboratory

[B17] Ruesch A Gibbs HK New IPCC Tier-1 Global Biomass Carbon Map For the Year 2000 2008 Oak Ridge, Tennessee: Carbon Dioxide Information Analysis Center, Oak Ridge National Laboratory

[B18] Kindermann GE McAllum I Fritz S Obersteiner M A global forest growing stock, biomass and carbon map based on FAO statistics Silva Fennica 2008 42 387 396

[B19] Le Toan T Ulander L Dubois-Fernandez P Papathanassiou K Rocca F Davidson M Retrieval of forest biomass from P-band SAR data: Prospects for the future BIOMASS mission Proceedings ESA Living Planet Symposium 2010 2010 Bergen, Norway

[B20] Drichi P National Biomass Study, Technical Report 2003 Forestry Department, Ministry of Water, Lands & Environment; PO Box 1613, Kampala, Uganda

[B21] Avitabile V Baccini A Friedl M Schmullius C Capabilities and limitations of Landsat and land cover data for aboveground woody biomass estimation of Uganda Remote Sensing of Environment in press

[B22] Olson JS Watts JA Allison LJ Major world ecosystem complexes ranked by carbon in live vegetation: A Database 1985 Oak Ridge, Tennessee: Carbon Dioxide Information Center, Oak Ridge National Laboratory

[B23] Mitchard ETA Saatchi SS Woodhouse IH Nangendo G Ribeiro NS Williams M Ryan CM Lewis SL Feldpausch TR Meir P Using satellite radar backscatter to predict above-ground woody biomass: a consistent relationship across four different African landscapes Geophysical Research Letters 2009 36

[B24] IPCC Guidelines for National Greenhouse Gas Inventories - Volume 4 - Agriculture, Forestry and other Land Use 2006 Institute for Global Environmental Strategies, Japan

[B25] Gaston G Brown S Lorenzini M Singh KD State and change in carbon pools in the forests of tropical Africa Global Change Biology 1998 4 97 114 10.1046/j.1365-2486.1998.00114.x

[B26] Van Vliet J Hagen-Zanker A Engelen G Hurkens J Vanhout R Uljee I Map Comparison Kit 3: User Manual 2009 Maastricht: Research Institute for Knowledge Systems

[B27] Hagen-Zanker A Comparing continuous valued raster data: A cross disciplinary literature scan 2006 Maastricht: Research Institute for Knowledge Systems

[B28] Woodcock CE Strahler AH Jupp DLB The use of variograms in remote sensing: I. scene models and simulated images Remote Sensing of Environment 1988 25 323 348 10.1016/0034-4257(88)90108-3

[B29] Woodcock CE Strahler AH Jupp DLB The use of variograms in remote sensing: II. real digital images Remote Sensing of Environment 1988 25 349 379 10.1016/0034-4257(88)90109-5

[B30] FAO Global ecological zoning for the global forest resources assessment 2000 FAO FRA Working Paper 2001 Rome, Italy

[B31] FAO Global forest resources assessment 2000 FAO Forestry Paper 2001 Rome, Italy

[B32] FAO Global forest resources assessment 2005 FAO Forestry Paper 2006 Rome, Italy

[B33] Brown S Gaston G Tropical Africa: Land Use, Biomass, and Carbon Estimates for 1980 1997 Oak Ridge, Tennessee: Carbon Dioxide Information Center, Oak Ridge National Laboratory

[B34] Lefsky MA A global forest canopy height map from the Moderate Resolution Imaging Spectroradiometer and the Geoscience Laser Altimeter System Geophysical Research Letters 2010 37

[B35] Saatchi SS Harris NL Brown S Lefsky M Mitchard ETA Salas W Zutta BR Buermann W Lewis SL Hagen S Petrova S White L Silman M Morel A Benchmark map of forest carbon stocks in tropical regions across three continents Proceedings of the National Academy of Sciences of the United States of America 2011 108 9899 9904 10.1073/pnas.1019576108 21628575 PMC3116381

[B36] FAO Multipurpose Africover Databases on Environmental resources http://www.africover.org/index.htm

[B37] Mayaux P Bartholome E Fritz S Belward A A new land-cover map of Africa for the year 2000 Journal of Biogeography 2004 31 861 877 10.1111/j.1365-2699.2004.01073.x

[B38] Arino O Bicheron P Achard F Latham J Witt R Weber JL GLOBCOVER The most detailed portrait of Earth Esa Bulletin-European Space Agency 2008 24 31

[B39] Hansen MC DeFries RS Townshend JRG Carroll M Dimiceli C Sohlberg RA Global Percent Tree Cover at a Spatial Resolution of 500 Meters: First Results of the MODIS Vegetation Continuous Fields Algorithm Earth Interactions 2003 7

[B40] Center for International Earth Science Information Network (CIESIN) and Centro Internacional de Agricultura Tropical (CIAT) Gridded Population of the World Version 3 (GPWv3): Population Density Grids 2005 Palisades, NY: Socioeconomic Data and Applications Center (SEDAC), Columbia University

[B41] Bryant D Nielsen D Tangley L The Last Frontier Forests: Ecosystems and Economies on the Edge 1997 Washington DC: World Resources Institute 42

[B42] Houghton RA The annual net flux of carbon to the atmosphere from changes in land use 1850-1990 Tellus Series B-Chemical and Physical Meteorology 1999 51 298 313 10.1034/j.1600-0889.1999.00013.x

[B43] DeFries RS Houghton RA Hansen MC Field CB Skole D Townshend J Carbon emissions from tropical deforestation and regrowth based on satellite observations for the 1980s and 1990s Proceedings of the National Academy of Sciences of the United States of America 2002 99 14256 14261 10.1073/pnas.182560099 12384569 PMC137871

[B44] Brown S Estimating biomass and biomass change of tropical forests: a primer FAO Forestry Paper 1997 Rome

[B45] Achard F Eva HD Stibig HJ Mayaux P Gallego J Richards T Malingreau JP Determination of deforestation rates of the world's humid tropical forests Science 2002 297 999 1002 10.1126/science.1070656 12169731

[B46] Achard F Eva HD Mayaux P Stibig HJ Belward A Improved estimates of net carbon emissions from land cover change in the tropics for the 1990s Global Biogeochemical Cycles 2004 18

[B47] Olson JS Watts JA Allison LJ Carbon in live vegetation of major world ecosystems 1983 Oak Ridge, Tennessee: Carbon Dioxide Information Center, Oak Ridge National Laboratory

